# *PRKN/PINK1* Mutations in a Chinese Patient With Early‐Onset Parkinson's Disease

**DOI:** 10.1002/brb3.70822

**Published:** 2025-09-02

**Authors:** Zhongjiao Lu, Ye Sun, Liemei Guo, Mei Xin, Yunlan Du, Ruolian Dai, Gang Chen, Hualong Wang, Gang Wang

**Affiliations:** ^1^ Department of Neurology, Renji Hospital, School of Medicine Shanghai Jiao Tong University Shanghai China; ^2^ Department of Neurosurgery, Renji Hospital, School of Medicine Shanghai Jiao Tong University Shanghai China; ^3^ Department of Nuclear Medicine, Renji Hospital, School of Medicine Shanghai Jiao Tong University Shanghai China

## Abstract

**Background:**

*PRKN* and *PINK1* gene mutations have been associated with Parkinson's disease (PD), particularly early‐onset PD (EOPD).

**Objectives:**

To describe the clinical and molecular features of a Chinese patient with EOPD who had uncommon *PRKN* and *PINK1* mutations.

**Methods:**

The patient's clinical history was reviewed, and whole‐exome sequencing was performed to identify genetic mutations. An in vitro mitochondrial stress model was created to study the impact of *PRKN* deletion on the PINK1‐PRKN pathway.

**Results:**

The patient exhibited a 27‐year history of tremors and other motor symptoms, with a notable response to levodopa and subthalamic nucleus deep brain stimulation (STN DBS). Genetic testing revealed an unusual double mutation of *PRKN/PINK1*, while *PRKN* deletion blocked the activation of the PINK1‐PRKN pathway, disrupting the patient's mitophagy pathway.

**Conclusions:**

*PRKN/PINK1* mutations may be linked to compromised mitophagy pathways. Genetic screening is significant for EOPD patients, especially those with specific symptoms and ethnic backgrounds.

## Graphical abstract

A 51‐year‐old Chinese man with 27‐year early‐onset Parkinson's disease harbored rare *PRKN/PINK1* mutations. PRKN deletion might abolish PINK1‐PRKN pathway activation, which is related to impaired mitophagy. Striatal dopaminergic dysfunction and levodopa‐responsive motor symptoms were reversed by subthalamic nucleus deep brain stimulation. Genetic screening is vital for EOPD management.

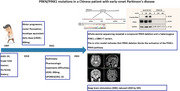

## 1 Introduction

Pathogenic variations in Parkinson's disease (PD)‐linked genes have been found in 5%–15% of patients. An earlier age at onset (AAO), a positive family history, and/or a specific heritage are all likely indicators of positive gene variations (Cook et al. 2024). However, the link between distinct genetic changes and the varied symptoms of PD remains unknown. The hidden and generic nature of initial symptoms delays diagnosis and challenges doctors in treating early‐onset PD patients (EOPD) (Mehanna et al. 2022).

## 2 Methods

**FIGURE 1:**
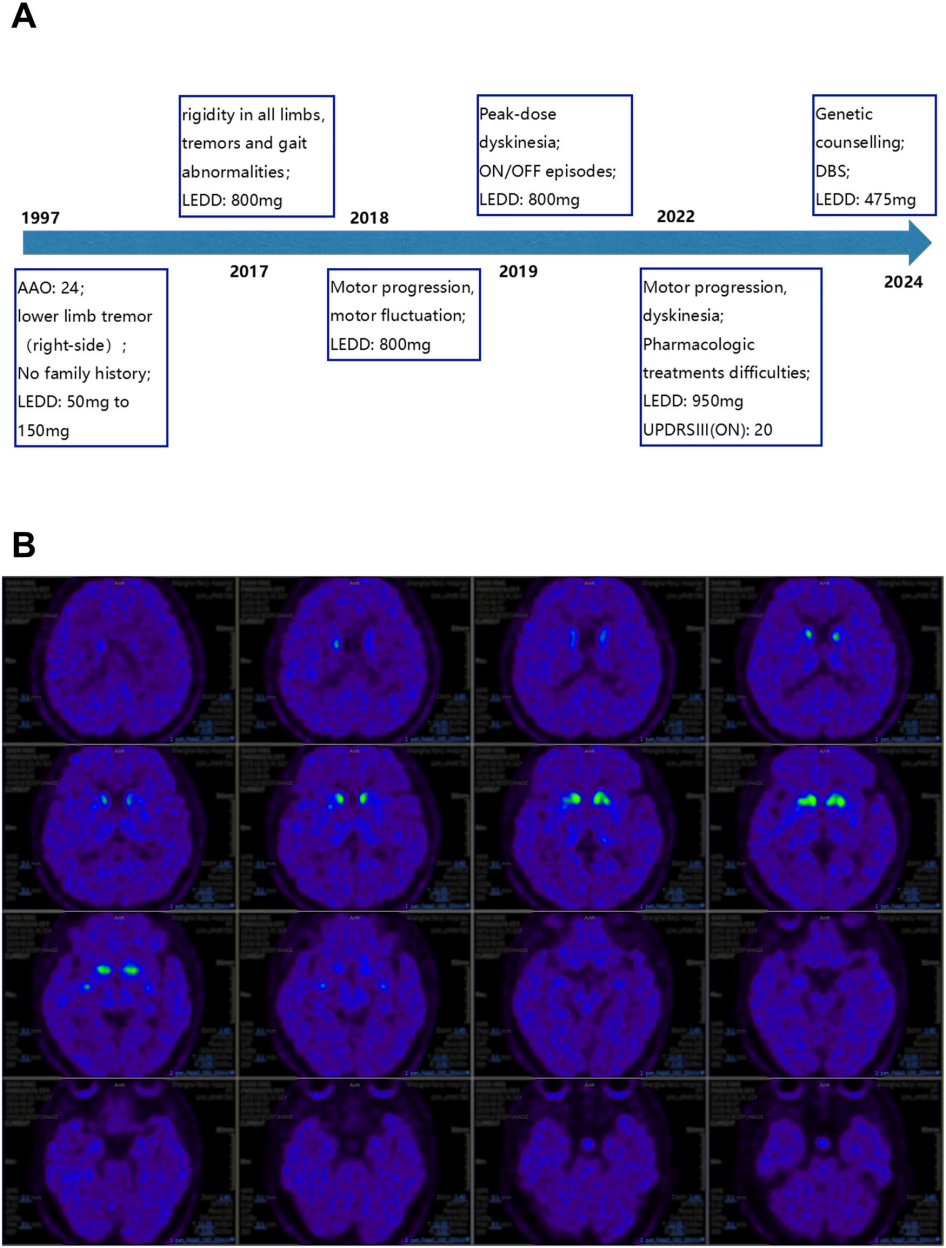
Disease Progression and Treatment of an EOPD Patient. (A) Disease progression: Before 2022, the patient took levodopa 200 mg four times daily; from 2022, 250 mg Sinemet per night was added. AAO: age at onset; LEDD: levodopa equivalent daily dose; DBS: deep brain stimulation, a surgical treatment method; UPDRS III: Unified Parkinson's Disease Rating Scale Part III, used to assess motor function. (B) PET scan: Reduced uptake of ^11^C‐CFT was observed in the bilateral caudate nuclei and putamina.

In August 2024, a 51‐year‐old male with a 27‐year history of tremors in his right lower limb presented to the movement disorders clinic at Renji Hospital (Figure 1A). He was born to healthy, normally developing, unrelated parents with a negative family history. Here, we went over his medical history, presented the patient's traits, and identified the associated pathogenesis using genetic testing, biochemical, imaging, and a cell model.

At the age of 24, he acquired resting and postural tremors in his right lower leg, accompanied by a forward‐leaning stride. With 50 mg of levodopa daily, symptoms improved. Over the next decade, his levodopa dose steadily climbed to 150 mg daily (levodopa equivalent daily dose, LEDD, 150 mg) (Jost et al. 2023), and his symptoms remained constant. In 2016, he experienced all‐limb rigidity, obvious tremors, and substantial gait problems. He then upped LEDD to 800 mg on his own, which relieved his symptoms. However, due to emotional turmoil caused by his father's death and a divorce, his symptoms changed. In 2018, he had increased limb rigidity, decreased medicine efficacy, and odd movements during dreams. Dyskinesias developed gradually, lasting about 4–5 h per day during his ON period. When instructed to reduce LEDD to 600 mg, he had uncontrollable tremor and rigidity; therefore, he returned to the previous dose of 800 mg. His dyskinesia worsened dramatically in 2022, and the medication's efficacy fell even more. He tried taking 250 mg of Sinemet at night and taking Pramipexole and Amantadine sporadically, but his symptoms did not get better. The patient has maintained levodopa and Sinemet at an LEDD of 950 mg for the past 2 years since he was unable to tolerate lowering the dosage.

## 3 Results

When he first came to us for his ON episode, he had somewhat increased muscle tone in his neck, left upper limb, and both lower limbs, as well as normal muscle strength and coordination. The flexibility of the right lower limb was reduced, as were resting tremors and postural tremors in both upper limbs. Dyskinesia was noticeable, and he walked with a downward tilt of his right shoulder (Video S1). His UPDRS scores (Goetz et al. 2008) of I–IV were 2, 5, 20, and 12, and his Montreal Cognitive Assessment (MoCA) was 21/30, showing modest cognitive loss, particularly in abstraction and delayed recall (Langa and Levine 2014). His other neuropsychological testing was normal and he responded with a 42.2% reduction in UPDRS III scores on the levodopa challenge test.

In terms of neuroimaging, the brain magnetic resonance imaging (MRI) scan returned normal results. A positron emission tomography (PET/MRI) scan, however, revealed increased FDG metabolism in the bilateral thalami and pons, while decreased metabolism was seen in the bilateral medial frontal lobes, bilateral inferior parietal lobes, right inferior frontal lobe, and bilateral lateral temporal lobes. The left dorsal frontal lobes were the most hypometabolic area. Reduced uptake of ^11^C‐CFT was observed in the bilateral caudate nuclei and putamina (Figure 1B), while the lowest standard uptake value ratio was found in the right posterior putamen (Table S1).

**FIGURE 2:**
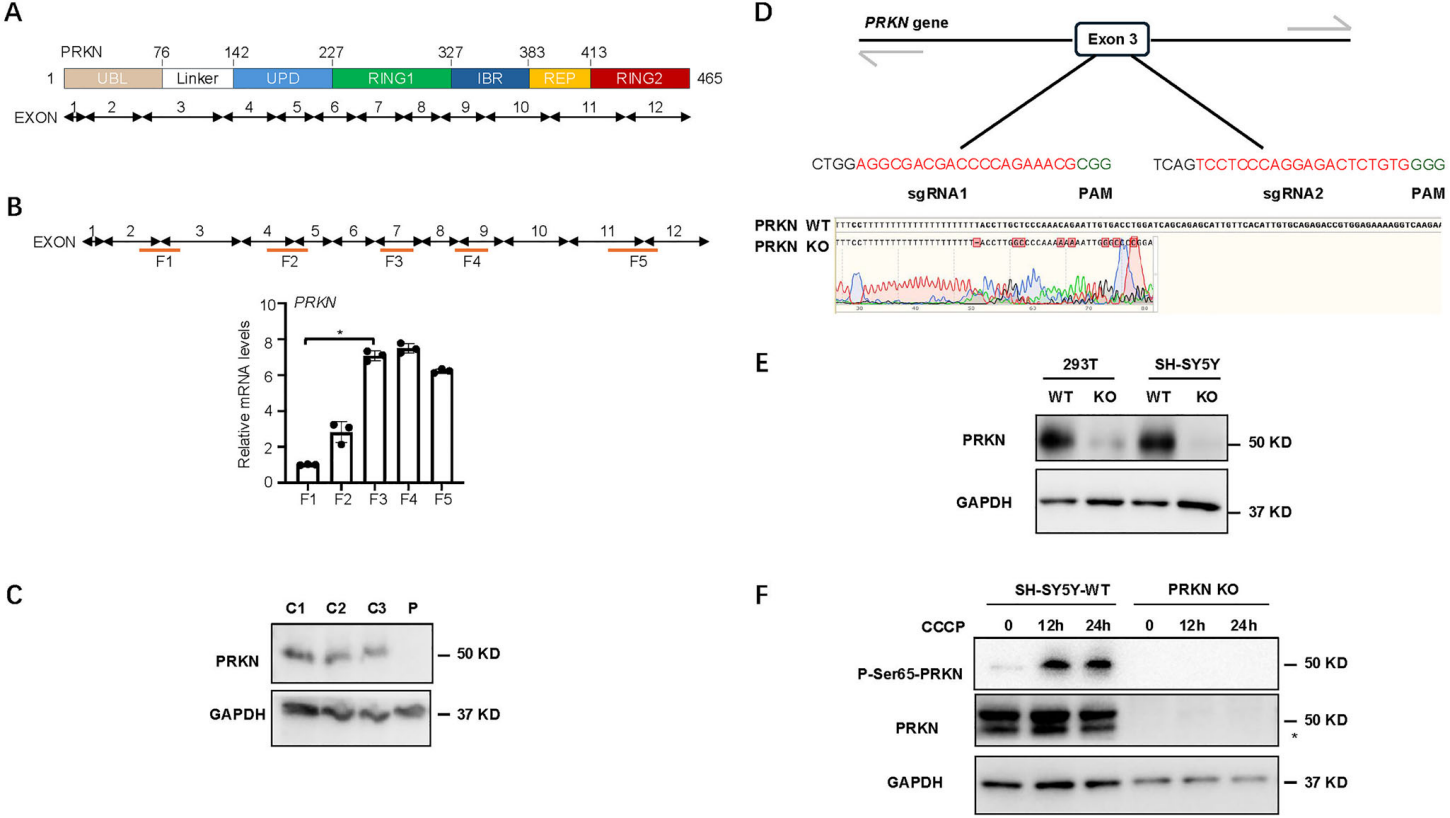
*PRKN* gene and protein expression. (A) Schematic diagram of PRKN protein functional domains. Human PRKN protein includes 465 amino acids (upper). Correspondingly, *PRKN* gene transcript has 12 exons (lower). (B) The regions of five pairs of primers are shown in red line under the 12 exons of *PRKN* gene transcript diagram, annotated with F1–F5 (upper). The relative mRNA expression level of five fragments in patient's PBMC is shown in the bar chart (lower). Data represent the mean ± SEM. (C) PRKN protein level in three control samples (C1–C3) and patient (P). PBMCs were isolated from three normal individuals and patient. (D) The *PRKN* knockout strategy is shown in 293T and SH‐SY5Y cell lines (upper); Sanger sequencing was used to detect the knockout fragment of *PRKN* in SH‐SY5Y cell line (lower). (E) PRKN protein knockout efficiency in 293T and SH‐SY5Y cell lines using western blot. (F) The PRKN phosphorylation of ser65 site was detected by western blot after 20 uM CCCP treatment under indicated conditions. The non‐specific band of PRKN is marked with a black asterisk.

Furthermore, whole‐exome sequencing identified a homozygous exon 3 deletion and a heterozygous exon 4 deletion in *PRKN*, as well as a heterozygous *PINK1* variant NM_032409.3:c.586C>T (p.P196S) in exon 2. To confirm gene expression, we created 5 distinct primer pairs between 12 *PRKN* exons (methods described in Supporting Information S1). The fragments 1 (F1) and 2 (F2) were reduced in the patient's peripheral blood mononuclear cells (PBMC), which was consistent with the genomic sequencing results (Figure 2B). The PRKN protein was undetectable in the patient's sample (P) compared to the three healthy control samples (C1–C3) (Figure 2C). Meanwhile, we knocked out *PRKN* using CRISPR‐Cas9 system by targeting exon3 area in 293T and SH‐SY5Y cells (Figure 2D,E) and created an in vitro mitochondrial stress model utilizing CCCP (carbonyl cyanide 3‐chlorophenylhydrazone) therapy at different times in SH‐SY5Y cell (Cook et al. 2024; Geisler et al. 2010). PRKN is downstream of kinase PINK1, so the phosphate of PRKN's serine 65 site increased in SH‐SY5Y WT cells, whereas PRKN and phosphorylated PRKN were undetectable in *PRKN* knock‐out SH‐SY5Y cells. The in vitro model indicates that PRKN deletion blocks the activation of the PINK1‐PRKN pathway, implying that our patient's mitophagy pathway has been disrupted as a result of PRKN deletion.

Therefore, the diagnosis is EOPD with *PRKN/PINK1* mutations. Subthalamic nucleus deep brain stimulation (STN DBS) surgery was proposed and carried out, resulting in significant symptom relief and a 50% reduction in dopaminergic medication (LEDD 950–475 mg) (Video S2).

## 4 Discussion

The case presented a *PRKN/PINK1*‐related EOPD with a lower AAO, slower development, early dyskinesia, and a favorable response to levodopa and STN DBS. Otherwise, it is puzzling that *PRKN* or *PINK1*‐PD caused different pathologies of dopaminergic neuronal loss and Lewy bodies, despite the fact that PRKN and PINK1 both sent disabled mitochondria to the lysosome for breakdown during mitophagy (Han et al. 2023).

Fewer studies have examined the relationship between the variations and structural/functional deficits to date, particularly when a patient has numerous mutations. Our case not only revealed unusual double mutations of *PRKN/PINK1* by WES, but also confirmed gene and protein expression (Figure 2). Only three examples in the Polish literature had previously been documented to have simultaneous mutations in the *PRKN/PINK1* genes; however, the mutation patterns differ from those seen in our cases (Milanowski et al. 2021). Furthermore, two other research works focusing on the North American and Chinese cohorts did not find similar multigene alterations (Cook et al. 2024; Sun et al. 2023). The patterns of multigene changes in *PRKN/PINK1* and their relationship with mitophagy require more investigation. Our findings indicated that the exon 3 deletion of *PRKN* in the patient, which was the main structural variant (located in the RING0 protein domain and the ubiquitin‐like protein domain), likely prevented its activation (Menon et al. 2024). The patient's motor symptoms are most likely caused by damaged mitophagy via the PINK1‐PRKN pathway (Geisler et al. 2010; Han et al. 2023). Our patient's unusual cognitive impairment could be attributed to FDG hypometabolism in the bilateral medial frontal and inferior parietal lobes, as seen in other cases with Lewy body illness (Ferreira et al. 2025). Our findings indicate that *PRKN/PINK1*‐PD is a hybrid of defective mitochondria and alpha‐synuclein aggregation, as previously shown in cell and animal models (Hou et al. 2023).

## 5 Conclusion

Genetic data are essential for creating gene‐specific trials because *PRKN/PINK1*‐PD has a better prognosis and an earlier AAO than sporadic PD. We propose providing a PD gene panel with *PRKN/PINK1* to EOPD patients, particularly those with AAO < 40 years of Asian heritage. More research is needed to confirm the phenotype–genotype links underlying the PINK1‐PRKN pathway and create gene‐specific treatments.

## Author Contributions

**Zhongjiao Lu**: conceptualization, funding acquisition, investigation, resources, writing – original draft, writing – review and editing. **Ye Sun**: investigation, resources, data curation, formal analysis, funding acquisition. **Liemei Guo**: conceptualization, investigation, resources. **Mei Xin**: investigation, resources. **Yunlan Du**: investigation, resources, data curation, formal analysis. **Ruolian Dai**: investigation, resources, data curation, formal analysis. **Gang Chen**: resources, investigation. **Hualong Wang**: methodology, writing – review and editing. **Gang Wang**: conceptualization, methodology, funding acquisition, writing – review and editing.

## Disclosure

The authors have nothing to report.

## Ethics Statement

The clinical study protocol and the informed consent form were reviewed and approved by the independent ethics committees of the participating sites. All participants gave full written consent, and the patient agreed to genetic analysis for diagnostic and scientific purposes and for online video publication. We confirm that we have read the Journal's position on issues involved in ethical publication and affirm that this work is consistent with those guidelines.

## Conflicts of Interest

The authors declare no conflicts of interest.

## Peer Review

The peer review history for this article is available at https://publons.com/publon/10.1002/brb3.70822.

## Supporting information



A. Detailed description of methods for confirming PRKN gene expression.
B. Table S1 The semi‐quantitative metrics extracted from ^11^C‐CFT PET data

Video: The patient's walking condition 3 h after taking levodopa medication before DBS surgery.

Video: Three months following DBS surgery, the patient's walking condition was assessed again three hours after taking levodopa, revealing a considerable reduction in walking tilt and tremors.

## Data Availability

The data that support the findings of this study are available from the corresponding author upon reasonable request.
