# Supplementary material for: PRKN/PINK1 Mutations in a Chinese Patient With Early‐Onset Parkinson's Disease
Source: Brain Behav. 2025 Sep 2;15(9):e70822. doi: 10.1002/brb3.70822 (PMC12405661; doi:10.1002/brb3.70822)
Supplement: Supplementary file 1 — A. Detailed description of methods for confirming PRKN gene expression. B. Table S1 The semi‐quantitative metrics extracted from 11C‐CFT PET data [file BRB3-15-e70822-s003.docx]

1. **Detailed description of methods for confirming PRKN gene expression.**

**QPCR and primers sequence**

Total RNA purification was performed using RNA purification kit (R2070, ZYMO) according to the manufacturer’s instructions. The reverse transcription of total RNA was performed using Thermoscript RT-PCR kit (Invitrogen) for further quantitative PCR (qPCR)(A314-10, Genstar). The primers used in qPCR are listed below:

|  | Forward | Reverse |
| --- | --- | --- |
| Fragment1(F1) | ACACCAGCATCTTCCAGCTC | TTTCTCCACGGTCTCTGCAC |
| Fragment2(F2) | CCGCCACGTGATTTGCTTAG | CCACACAAGGCAGGGAGTAG |
| Fragment3(F3) | AACTCAGGGTACAGTGCAGC | TCACCACTCATCCGGTTTGG |
| Fragment4(F4) | GTACAACCGGTACCAGCAGT | CCTTCGCAGGTGACTTTCCT |
| Fragment5(F5) | AGAGTCGATGAAAGAGCCGC | CTTCATGTGCATGCAGCCTC |

**WB and antibody**

Cell lysates were prepared using RIPA lysis buffer (50 mM Tris-HCl pH 8.0, 150 mM NaCl, 5 mM EDTA, 0.1% SDS and 1% NP-40) together with phosphatase (Roche) and proteinase (Sigma) inhibitor cocktails. Extracted proteins were boiled at 100 °C for 10 min and then subjected to electrophoresis through 10% SDS–polyacrylamide gel electrophoresis. GAPDH (1:1000) antibody was purchased from Servicebio (GB15004-100); PRKN(1:1000) antibody was purchased from zenbio(381626); p-Ser65-PRKN(1:1000) was purchased from abcam(ab315376).

**Knock Out with CRISPR-CAS9**

Construct plasmids comprise 2 sgRNA targeting PRKN exon3 (target sequence:1. AGGCGACGACCCCAGAAACG; 2. TCCTCCCAGGAGACTCTGTG) with PX330 backbone.

Transient transfection of 2 plasmids into 293T and SH-SY5Y cell lines respectively using lipofectamine 2000(invitrogen,11668019). The puromycin-resistant cells were selected for 48 h by 2ug ul-1 puromycin (P8230, Solarbio) at 24 h post-transfection. The puromycin-resistant cells were then sorted into 96-cell- plates with one cell in every well. The knockout result was confirmed with PCR and sanger sequencing.

**Human PBMC isolation**

Fresh peripheral blood was first centrifuged at 2000rpm in room temperature. Remove upper plasma layer, mix the remaining red blood cells with same volume PBS. Slowly drip the mixture into the top of Ficoll solution (Cytiva, 17544202). Centrifuge for 30min at 800g in room temperature with accel 1 and brake 1. Transfer PBMC cells between the plasma layer and the Ficoll layer into a new 15ml tube. Wash PBMC with sterile PBS 3 times.

**Cell culture**

HEK293T and SH-SY5Y cells were grown in a DMEM medium supplemented with 10% FBS (FCS500, ExCell Bio) and 1% penicillin/streptomycin (Invitrogene). All cells were maintained at 37°C and 5% CO2 under humidified conditions. Cells are passaged every 2 days. The mycoplasma contamination was tested every month. All cells were mycoplasma negative.

**Statistical analysis**

Data are expressed as the mean ± SEM. Data were analyzed using a two-tailed unpaired Student’s t-test (GraphPad Prism software, version 10.01). p < 0.05 was considered statistically significant marked with a black asterisk in figure.

1. **Table S1**

| Table S1 The semi-quantitative metrics extracted from ^11^C-CFT PET data | | | |
| --- | --- | --- | --- |
|  |  | SUVr | AI |
| Caudata | Right | 1.339 | 0.00 |
|  | Left | 1.373 |  |
| Anterior Putamen | Right | 0.782 | 0.21 |
|  | Left | 1.056 |  |
| Posterior Putamen | Right | 0.498 | 0.13 |
|  | Left | 0.562 |  |

SUVr: Standard uptake value ratio, calculated by striatal standard uptake value (SUV) to occipital SUV ratio; AI: Asymmetry Index
